# Research Progress on the Regulation of Plant Floral Organ Development by the *MADS-box* Gene Family

**DOI:** 10.3390/ijms26188946

**Published:** 2025-09-14

**Authors:** Qiufei Wu, Yi Wu, Rui Li, Hongxing Cao, Zongming Li, Qihong Li, Lixia Zhou

**Affiliations:** 1 State Key Laboratory of Tropical Crop Breeding, Chinese Academy of Tropical Agricultural Sciences, Sanya 572024, China; 2 Coconut Research Institute, Chinese Academy of Tropical Agricultural Sciences, Wenchang 571339, China

**Keywords:** floral initiation and development, *MADS-box* genes, floral development models, regulatory mechanisms

## Abstract

The initiation, development, and morphological construction of floral organs constitute a highly intricate process, involving numerous factors and their interactions. *MADS-box* genes are key regulators of developmental processes and are consequently the most extensively studied gene family in floral organ research. By synthesizing current understanding of the regulatory roles of *MADS-box* genes in the initiation, differentiation, and morphogenesis of floral organ, this review provides novel insights into the floral development program and the general transcriptional regulatory mechanisms of this gene family. It also offers a reference for further in-depth exploration of this gene family and the refinement of theories governing floral development regulation.

## 1. Introduction

Floral organs are essential for the reproduction in angiosperms and represent one of the most morphologically diverse organ systems in plants. In response to varying in growth environments and reproductive strategies, these organs have evolved into a wide array of forms that enhance reproductive success. While some plants, such as those in the *Orchidaceae* family and *Snapdragons*, exhibit highly specialized and unique floral morphologies, the majority of angiosperms possess a typical four-whorled structure, consisting from the outside inward of sepals, petals, stamens, and carpels. Floral organs serve as vital indicators for studying plant evolution and classification and have consistently been a focal point of research. Since the cloning of the first *MADS-box* gene associated with floral development from the model plant *Arabidopsis thaliana*, studies on the regulation of floral development and related genes have advanced significantly. Molecular-level research has revealed that the initiation, differentiation, and formation of floral organs are governed by a coordinated mechanism involving the transcriptional regulation and interaction of various genes, within which the *MADS-box* gene family plays a pivotal regulatory role [1].

*MADS-box* genes encode transcription factors that are ubiquitously present in all eukaryotes [2], influencing the morphogenesis and growth of various plant organs, such as roots and fruits [3,4]. The in-depth study of *MADS-box* genes has been propelled by their crucial functions in floral organs. The term “MADS” is derived from the initial letters of four genes: *Minichromosome maintenance* gene (*MCM1*) in yeast, *AGAMOUS* (*AG*) in *Arabidopsis thaliana*, *DEFICIENS (DEF*) in snapdragon, and the human *SerumResponse Factor* (*SRF*). The above four genes all contain a highly conserved region known as the MADS-box domain, located near the N-terminus. This domain consists of approximately 180 base pairs and encodes a DNA-binding motif that recognizes similar target DNA sequences [5]. To date, research on the functions of *MADS-box* genes in plants has been extensive, with numerous *MADS-box* genes identified and their functions validated in model plants such as *Arabidopsis thaliana*, *snapdragon*, *petunia*, rice, and maize [6]. However, the expression patterns of *MADS-box* genes are not entirely consistent across different plant species, necessitating further research to explore the model systems of floral organ development in various plants through the functional analysis of homologous genes. This article summarizes recent advances in the regulation of floral organ initiation and development by *MADS-box* genes, elucidates the regulatory network mechanisms underlying floral development, and provides a foundation for further exploration of *MADS-box* homologous genes and protein function analysis. This will contribute to refining the regulatory network of floral development and investigating the model systems of floral development in different plant species.

## 2. Classification and Structure of *MADS-box* Genes

Early phylogenetic analyses classified *MADS-box* genes into distinct subfamilies according to their functions. Subsequent comparative genomic studies, primarily using *Arabidopsis thaliana* sequences, established the phylogenetic relationships among major evolutionary branches of this gene family and confirmed that a single ancestral duplication event gave rise to two lineages (Type I and Type II) in plants, animals, and fungi [7]. In plants, Type I genes are phylogenetically clustered with *SRF-like* genes from animals. Structurally, Type I genes typically contain only 1–2 exons and encode proteins featuring a conserved core MADS-domain and a highly variable Carboxy-terminal domain [8]. Owing to these structural characteristics and their functional importance, Type I genes have attracted growing research interest [9]. For example, several Type I genes cloned from *Arabidopsis thaliana*, including *AGL23*, *AGL28*, *AGL61*, *AGL62*, *AGL37*, and *AGL80*, play crucial roles in endosperm development and are essential for female gametophytes and embryo formation [10,11]. Similarly, in *Moso bamboo*, six Type I genes have been implicated in inflorescence development, with *PeMADS5* promoting early flowering when heterologously expressed in *Arabidopsis thaliana* [12].

**Figure 1:**
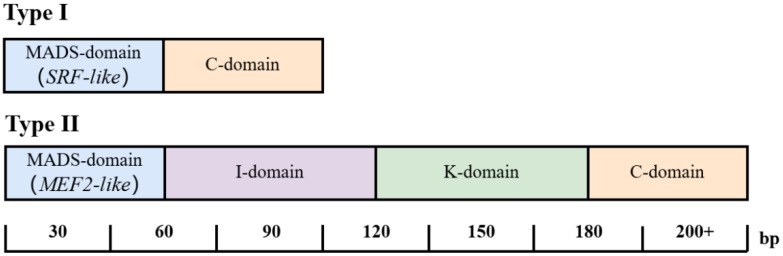
Classification and structure of MADS-box proteins. I-domain means intervening domain, K-domain means keratin-like domain, and C-domain means C-terminal domain.

In angiosperms, the majority of *MADS-box* genes identified belong to the Type II lineage. Type II genes are further divided into two branches: *MEF2*-type, primarily found in animals and fungi, and *MIKC*-type, which is unique to plants. *MIKC*-type genes typically consist of six introns and seven exons, encoding proteins composed of four domains: the MADS-domain, I-domain (intervening domain), K-domain (keratin-like domain), and C-terminal domain (Figure 1). The K-domain is a characteristic feature of *MIKC*-type genes, while the C-terminal domain is the least conserved region, with variations in its structure potentially leading to functional differences among MADS-box proteins. Research indicates that the I and K domains are involved in protein–protein interactions of *MADS-box* transcription factors, while the C-terminal domain, acting as a transcriptional activation domain, may play a role in stabilizing these interactions [13,14]. The *MIKC*-type is further subdivided into *MIKCC*-type and MIKC*-type, with the *MIKCC*-type constituting the majority and encompassing all known plant *MADS-box* genes with characterized expression patterns or mutant phenotypes [15] (Table 1).

## 3. Regulation of Floral Organ Development by the *MADS-box* Gene Family

From cytological and anatomical perspectives, floral development initiates with the transformation of meristematic cells, a process driven by both endogenous and environmental factors. This transformation initiates flowering, during which cells transition from the shoot apical meristem (SAM) to the inflorescence meristem (IM) [16]. The IM then generates floral meristems at its periphery, which subsequently differentiate into distinct floral organ whorls. In monocots, the IM first produces spikelet meristems, which then differentiate into floral meristems [2,17]. This developmental cascade is tightly regulated by a complex network of factors, including flowering integrators (*FT*, *SOC1*), floral meristem identity genes (*AP1*, *LFY*, *CAL*, *FUL*, *AGL24*), and floral organ identity genes (ABCDE-class genes), alongside other regulatory elements. The morphological patterning of floral organs is orchestrated by multiple signaling pathways and gene families, with key determinants shaping phenotypic variation. As central regulators, *MADS-box* genes exhibit differential expression levels that specify distinct morphological structures. These genes encode transcription factors that modulate floral architecture through DNA binding or protein–protein interactions. Additionally, the responsiveness of downstream target genes further contributes to the diversity of floral organ phenotypes.

### 3.1. Role of MADS-box Genes in Floral Initiation

The initiation of flowering is a process driven by signal transduction and the transformation of plant cell identity. To date, six major flowering signaling pathways have been characterized: the photoperiod pathway, vernalization pathway, autonomous pathway, gibberellin pathway, temperature-sensitive pathway, and age pathway [2]. These pathways perceive and relay flowering signals, ultimately regulating two antagonistic classes of *MADS-box* genes that control flowering onset. One class consists of strong flowering repressors, including *FLOWERING LOCUS C* (*FLC*) and *SHORT VEGETATIVE PHASE* (*SVP*), while the other comprises flowering integrators and floral meristem identity genes, which promote floral bud differentiation. The vernalization, autonomous, and temperature-sensitive pathways primarily suppress *FLC* and *SVP* expression through post-transcriptional regulation and epigenetic modifications (e.g., histone methylation), thereby alleviating their repression of flowering. Notably, *FLC*, a *MADS-box* transcription factor, directly inhibits the transcription of flowering integrators *FT* and *SOC1* to delay flowering [18,19]. Prior to flowering, *FLC* exhibits high baseline transcriptional levels. Upon receiving flowering initiation signals, *FRI*, histone acetyltransferases, the histone methyltransferase COMPASS-like, and other chromatin modifiers are part of a FRI-containing supercomplex enriched in a region around the *FLC* transcription start site (TSS) to promote the expression of *FLC* antisense mRNA, leading to a reduction in sense mRNA expression. Concurrently, chromatin-modifying complexes alter the chromatin state of the *FLC* locus through histone modifications, further repressing *FLC* expression [20,21]. In the temperature-sensitive pathway, high temperatures promote the expression of FT while suppressing *SVP*, which interacts with *FLC* to negatively regulate the transcriptional activity of flowering integrators [22], thereby influencing flowering. However, the precise molecular mechanisms remain unclear. Interestingly, *SVP* and the floral meristem identity gene *AGL24* are paralogous genes within the *SVP*/*AGAMOUS*-LIKE lineage of the *MADS*-*box* gene family. While *SVP* inhibits floral organ formation, *AGL24* acts as a flowering activator [23]. In the monocot wheat, the *SVP* ortholog *TaVRT2*, along with *TaVRN1*, functions as a flowering initiator in the vernalization pathway. The *TaVRT2* protein can directly bind to the promoter of *TaVRN1*, mediating a positive feedback loop [24,25], which differs from the regulation of *SVP* in dicots. Therefore, further research is needed to elucidate the regulatory roles of *SVP* and its orthologs in floral organ formation. In the photoperiod, gibberellin, and age pathways, signals directly or indirectly activate the expression of genes such as *FT*, *SOC1*, *AP1*, and *AGL24* to initiate flowering [26,27,28].

### 3.2. Regulation of Cell Differentiation by MADS-box Genes

The six major flowering signaling pathways converge on two central integrators: *FLOWERING LOCUS T* (*FT*) and *SUPPRESSOR OF OVEREXPRESSION OF CONSTANS 1* (*SOC1*). While *FT* is not a *MADS-box* gene, it encodes a mobile protein that translocates over long distances to the shoot apex. There, it activates the downstream floral meristem identity gene *AP1*, initiating floral meristem formation at specific positions on the inflorescence meristem [29]. *SOC1* serves as a hub that integrates flowering signals from multiple pathways. It forms a self-regulatory feedback loop with *AGL24* through reciprocal promoter binding. Furthermore, the *SOC1*-*AGL24* heterodimer cooperatively upregulates *LEAFY* (*LFY*) expression [30], a critical regulator of floral development. Within the shoot apical meristem (SAM), the concerted upregulation of *SOC1* and other factors drives the transition from vegetative growth to inflorescence meristem identity [31].

The formation of floral meristems requires the decisive action of floral meristem identity genes (*AP1*, *LFY*, *CAL*, *FUL*, *AGL24*), all of which, except *LFY*, are *MADS-box* genes. *APETALA1* (*AP1*) and *CAULIFLOWER* (*CAL*) are paralogous genes whose high expression determines the differentiation of floral meristems. In *Arabidopsis*, the ap1-cal double mutant exhibits prolific shoot proliferation at single flower positions, a phenomenon known as the “cauliflower phenotype,” where the inflorescence meristem fails to differentiate into floral meristems [32]. Although *FRUITFULL* (*FUL*) is expressed only in the inflorescence meristem and not in the floral meristem, it plays a crucial role in the initiation and differentiation of floral meristems. In *Arabidopsis*, *FUL* and *AP1* exhibit functional redundancy in determining floral meristem identity [33].

*MADS-box* transcription factors play a pivotal role in orchestrating floral development by regulating stage-specific transitions between meristem identities. They achieve this by dynamically modulating the expression of identity genes, thereby establishing new developmental programs while repressing previous ones. The shift from vegetative growth to inflorescence meristem (IM) identity is governed by flowering integrators, which assimilate signals from multiple pathways to initiate this transition. Subsequently, integrators such as *SOC1* activate floral meristem identity genes, promoting the formation of floral meristems on the flanks of the IM while simultaneously suppressing IM identity genes [34]. Research has demonstrated that *SOC1*, *AGL24*, and *AGL15* interact with chromatin complexes to suppress premature activation of the floral organ identity gene *SEP3*, thereby preventing the early differentiation of floral meristem cells [35]. Once the floral meristem is established, *AP1* gradually downregulates earlier developmental programs, including its own activators such as *SOC1* [36]. Furthermore, *AP1* upregulates *LFY*, and together, these genes play a crucial role in floral organ specification. *AP1* also forms dimers with SEP3, an E-class protein of the *MADS*-*box* gene family, to activate genes responsible for floral organ identity [37].

### 3.3. Regulation of Floral Organ Formation by MADS-box Genes

**Figure 2:**
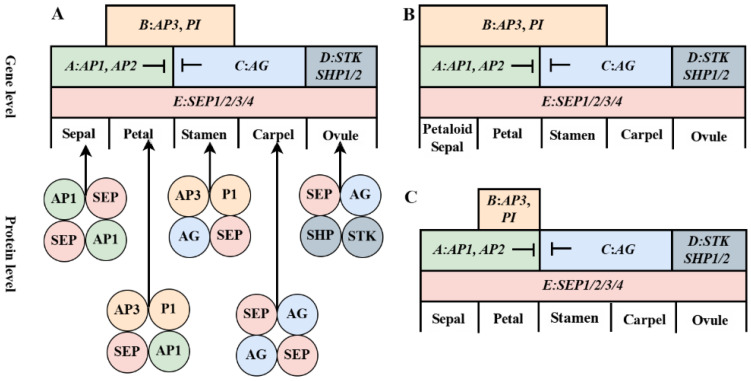
Floral organ development models. (**A**): Interpreted floral development models at different levels. “The ABC (D) E model” was at gene level. Sepals: A-class genes alone specified; Petals: A-and B-class genes combined to specified; Stamens: B-and C-class genes combined to specified; Carpels: C-class genes alone specified; Ovules: D-class genes specified (partially involved C-class genes); E-class genes were required to co-regulate the formation of floral organs. “The quartet model” was at protein level. Tetramers were formed by homodimer or heterodimer. Sepals: AP1-SEP-AP1-SEP; Petals: AP3-PI-SEP-AP1; Stamens: AP3-PI-SEP-AG; Carpels: AG-SEP-AG-SEP; Ovules: AG-SEP-SHP-STK. (**B**): Petaloid sepals in Liliaceae of monocotyledon resulted as B-class genes which covered the first three whorls. (**C**): B-class genes were expressed only in parts of the petals in some *Ranunculus*. *AP2* gene was not a *MADS-box* gene; the other ABCDE genes were all *MADS-box* genes. *AP1*: *APETALA1*, *AP2*: *APETALA2*, *AP3*: *APETALA3*, *PI*: *PISTILLATA*; *AG*: *AGAMOUS*, *STK*: *SEEDSTICK*, *SHP1*: *SHATTERPROOF1*, *SHP2*: *SHATTERPROOF2*, *SEP*: *SEPALLATA*.

**Figure 3:**
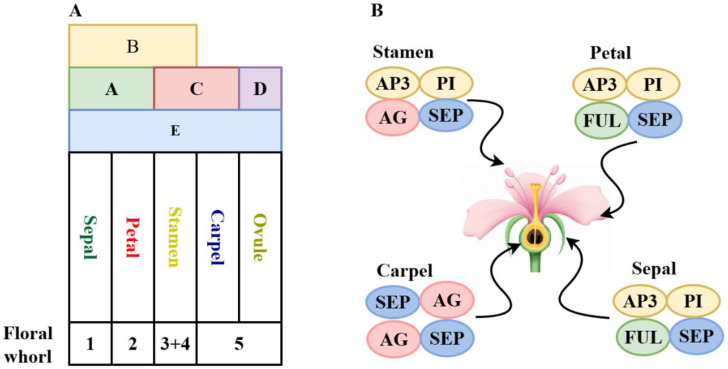
ABCDE model of floral development (**A**) and floral quartet model (**B**). *AP1*: *APETALA1*, *AP2*: *APETALA2*, *AP3*: *APETALA3*, *PI*: *PISTILLATA*; *AG*: *AGAMOUS*, *STK*: *SEEDSTICK*, *SHP1*: *SHATTERPROOF1*, *SHP2*: *SHATTERPROOF2*, *SEP*: *SEPALLATA*.

Under the regulation of floral organ identity genes (ABCDE-class genes), cells of the floral meristem sequentially form different floral organs from the outer to the inner whorls along the flanks of the primordia. With the exception of the A-class gene *AP2*, which belongs to the *AP2/ERF* family, all floral organ identity genes are *MADS-box* genes. For most of these genes, their expression domains reflect their functional domains. A-class genes are expressed in sepals and petals, where they independently regulate sepal formation and synergistically regulate petal development with B-class genes. B-class and C-class genes are co-expressed in stamens to regulate their development and differentiation, while C-class and D-class genes regulate the formation of carpels and ovules. A-class and C-class genes exhibit antagonistic interactions, mutually restricting their expression domains within the floral primordia, thereby defining the boundaries of floral organs [37]. When A-class genes are mutated, C-class genes are expressed in the first and second whorls (sepals and petals), and vice versa, a phenomenon explained by the classic “ABC model of floral development” (Figure 2A). This model has been used to analyze the correspondence between the floral structures of monocots and the floral organs of dicots, providing evidence that the lemma and palea are homologous to sepals, and the lodicules are homologous to petals [38]. Ectopic expression of these genes can lead to alterations in floral organ structure, as seen in the triple mutants of *Arabidopsis*. In nature, floral organ structures vary widely. For example, in monocots such as lilies, the petaloid sepals in the first whorl result from the ectopic expression of B-class genes in the outer three whorls (Figure 2B), and reducing the expression of B-class genes affects the formation of floral perianth characteristics [39]. In some basal eudicots, such as those in the *Ranunculaceae*, B-class genes are expressed only in certain petals [36] (Figure 2C). With further research into floral development across different plants, the more complex ABCDE model has gained widespread acceptance (Figure 3). Studies on petunias introduced the D-class genes *FLORAL BINDING PROTEIN7* (*FBP7*) and *FLORAL BINDING PROTEIN11* (*FBP11*), with their homologs in *Arabidopsis* being *SEEDSTICK* (*STK*) and *SHATTERPROOF* (*SHP*), which are responsible for ovule determination and formation [40,41]. Meanwhile, E-class genes, such as *SEPALLATA* (*SEP*), are expressed throughout the floral meristem and are essential for the proper formation of all floral organs. SEP proteins can form multimeric complexes with A, B, and C-class proteins, maintaining their normal functions [42,43].

The expression levels of *MADS*-*box* genes regulate the morphological formation of floral organs, with biochemical regulation involving the binding of transcription factor-encoded proteins to target DNA sequences or interactions with other proteins. *MADS*-*box* transcription factors regulate complex gene networks in plants, requiring high specificity to target different genes. All *MADS*-*box* transcription factors recognize the CArG (CC[A/T]6GG) motif on DNA as dimers. Specifically, MIKC^C^-type MADS-box proteins can bind DNA at two sites as dimers and then form tetramers, causing DNA looping and structural changes to either inhibit or activate target gene expression. This regulatory mechanism is unique to plants [8]. For example, B-class gene proteins such as AP3 and PI bind DNA exclusively as heterodimers and maintain their transcription through a self-regulatory feedback loop involving their own promoters [44]. Yeast two-hybrid experiments have shown that the heterodimers formed by B-class orthologs DEF and GLO exhibit strong DNA-binding activity. Meanwhile, E-class gene-encoded proteins function as cofactors by forming complexes that help activate ABC genes and interact with their homo- or heterodimeric proteins to form tetramers, regulating floral organ formation. This is the basis of the tetramer model of regulation [45] (Figure 2A). Different MADS-box proteins vary in their ability to activate target gene transcription. For instance, the AP3-PI heterodimer binds to the CArG-box in the *AP3* promoter but cannot activate transcription unless it forms a trimer with AP1 or SEP3 proteins, which then activates the *AP3* gene promoter [46].

### 3.4. Research Progress on ABCDE-Class Genes

#### 3.4.1. A-Class Genes

In *Arabidopsis*, class A genes include *AP1* and *AP2*. Among these, only *AP1* contains a conserved MADS domain and encodes a *MADS-box* transcription factor. *AP1* is specifically expressed in floral tissues, not only in the first whorl (sepals) and the second whorl (petals), but also plays a critical role in establishing floral meristem identity during early flower differentiation [47,48,49]. In situ hybridization analyses reveal that *AP1* expression is initiated in floral primordia and gradually intensifies as the primordia develop. Although not strictly essential for floral meristem formation, its expression is sufficient to convert an inflorescence into a flower [50]. The *ap1* mutant in *Arabidopsis* produces leaf-like bracts in place of sepals and exhibits an almost complete loss of petals [51]. Furthermore, primary *ap1* flowers often show partial conversion of floral organs into ectopic secondary inflorescences [52]. These observations demonstrate that *AP1* regulates both floral meristem identity and the development of the first- and second-whorl floral organs. Besides *AP1*, the *Arabidopsis* genome contains three other AP1-subfamily members: *CAULIFLOWER* (*CAL*), *FRUITFULL* (*FUL*), and *AGL79*. Among them, *CAL* and *FUL* are paralogs of *AP1*, and all three genes cluster phylogenetically within a group named after *SQUA*, the snapdragon ortholog of *AP1* [52]. Double mutants of *ap1* and *cal* exhibit a “cauliflower” phenotype, in which positions normally occupied by individual flowers are replaced by proliferating masses of inflorescence meristems [53]. This phenotype is enhanced in the *AP1 CAL FUL* triple mutant, indicating a synergistic role among these genes [54]. The mutant analyses confirm that *AP1*, *CAL*, and *FUL* collectively act as floral meristem identity genes. In dicot plants, *AP1*, *CAL*, and *FUL* together fulfill the functions of class A genes [55], whereas in monocots, only *FUL* is associated with class A gene functions [56].

As the only gene in the ABCDE model that does not belong to the *MADS-box* family, *AP2* possesses a unique AP2/ERF-type domain and encodes a transcription factor within the AP2/ERF family. The AP2 domain, comprising approximately 60–70 amino acids, is highly conserved and forms an amphipathic α-helix. It mediates protein–protein interactions, contains nuclear localization signals, and functions in transcriptional regulation [57]. AP2/ERF transcription factors can be categorized into three subfamilies based on the number of AP2 domains. Proteins with two AP2 domains belong to the AP2 subfamily, which is further divided into two groups: the AP2 group and the ANT (AINTEGUMENTA) group. Most members of this subfamily are involved in plant developmental processes, including floral organ morphogenesis, inflorescence meristem formation, and ovule and seed development. In *Arabidopsis*, the AP2 group includes five genes: *AP2*, *TOE1*, *TOE2*, *SHLAFMUTZE* (*SMZ*), and *SCHNARCHZAPFEN* (*SNZ*). The ANT group consists of *ANT*, *AIL1*, *AIL5*, *AIL6*, *AIL7*, *AtBBM*/*AIL2*, *PLETHORA1* (*PLT1*), and *PLETHORA2* (*PLT2*)/*AIL4* [58]. *AP2* is a key transcription factor required for establishing floral meristem identity, determining floral organ identity, and regulating the expression of homeotic genes. It is expressed in both floral and non-floral tissues, with distinct temporal and spatial patterns [59]. Weak *AP2* mutants exhibit homeotic transformations—sepals become leaf-like and petals develop as stamen-like structures [60]—while strong *ap2* mutants show a conversion of first-whorl organs into carpels and a reduction in organ number in the second and third whorls [61]. These phenotypes confirm that *AP2* plays a critical role in regulating floral development in the first two whorls, and its loss leads to ectopic expression of *AG*, consistent with the ABC model. Studies of other AP2 subfamily genes in *Arabidopsis* indicate that *TOE1* and *TOE2* act as floral repressors [62], *SMZ* and *SNZ* are involved in flowering time control [63], and *ANT* is essential for ovule and female gametophyte development, partially overlapping in function with *AP2* [64,65,66]. In petunias, the gene with the highest homology to *Arabidopsis AP2* is *PhAp2A*, which exhibits similar spatiotemporal expression and functional characteristics. In snapdragons, *LIP1* and *LIP2* serve as functional equivalents of *AP2* and contribute to sepal, petal, and ovule development. However, unlike AP2, they do not repress the expression of C-class genes in floral organs.

#### 3.4.2. B-Class Genes

*Arabidopsis thaliana* possesses two class B floral organ identity genes, *AP3* and *PI*, which belong to the *DEF* and *GLO* clades, respectively—named after their orthologs in *Antirrhinum majus* [67]. Mutations in these genes result in the replacement of petals (second whorl) by sepals and the transformation of stamens (third whorl) into carpel-like structures [67]. These findings demonstrate that class B genes are not only essential for specifying petal identity but also play a critical role in plant sex determination by promoting stamen development and suppressing carpel formation. Evolutionary studies indicate that the function of class B genes in specifying reproductive organ identity is highly conserved across angiosperms and even among seed plants more broadly [68]. However, gene duplication events during angiosperm evolution have given rise to distinct *DEF* and *GLO* lineages, leading to partial subfunctionalization and neofunctionalization. Notably, the encoded proteins have evolved from ancestral homodimers into obligate heterodimers. In orchids, the expression of class B genes extends into the first whorl of floral organs, contributing to their characteristic petaloid perianth. The remarkable diversity and specialized morphology of orchid flowers may also reflect evolutionary innovations within the class B gene family [45].

#### 3.4.3. C- and D-Class Genes

The *AG* gene, a canonical class C gene in *Arabidopsis thaliana*, plays a central role in regulating the development of stamens, carpels, ovules, and fruits, and is crucial for floral organ formation and differentiation. Studies on the *Arabidopsis ag* mutant show that while sepals and petals develop normally, the stamens in the third whorl are transformed into petals, and the fourth whorl produces a new *ag* flower instead of carpels—consistent with predictions from the floral organ development model. Additionally, *AG* represses the expression of *AP1* in the third and fourth whorls, although *AP1* does not reciprocally inhibit *AG*. Instead, *AG* expression is suppressed in the first and second whorls by *AP2* and five other genes in *Arabidopsis* [69]. Takeda et al. (2022) identified a new repressor of *AG*, *RABBITEARS* (*RBE*), which is involved in petal development and, like *ANT*, suppresses *AG* expression in the second whorl [70]. Class D genes are responsible for specifying ovule identity during floral morphogenesis. Pinyopich et al. (2003) demonstrated that *SHP1*, *SHP2*, and *STK* all contribute to ovule development [71]. Single and double mutants of these genes developed normal ovules, whereas triple mutants exhibited a transformation of ovules into leaf-like or carpel-like structures. Ectopic expression of any one of these three genes resulted in the formation of sepal–ovule chimeric structures. In addition to their role in ovules, *SHP1* and *SHP2* also regulate carpel development [72].

#### 3.4.4. E-Class Genes

Class E genes encode a group of functionally essential transcription factors that are required for the development of all types of floral organs. Their protein products interact with class A, B, C, and D floral homeotic proteins to form multimeric MADS-box complexes, which are critical for normal plant growth and floral organ differentiation [73]. In *Arabidopsis thaliana*, four *SEP* genes (*SEP1*, *SEP2*, *SEP3*, and *SEP4*) have been identified. The *sep1/2/3* triple mutant exhibits homeotic transformations in which petals, stamens, and carpels are converted into sepal-like organs—a phenotype resembling that of class B and C double mutants [44]. Notably, the expression of class A, B, and C genes remains unchanged in these mutants. These results confirm that class E genes are necessary for maintaining floral organ and meristem identity and exhibit partial functional redundancy.

### 3.5. Regulation of Downstream Target Genes by MADS-box Genes

**Figure 4:**
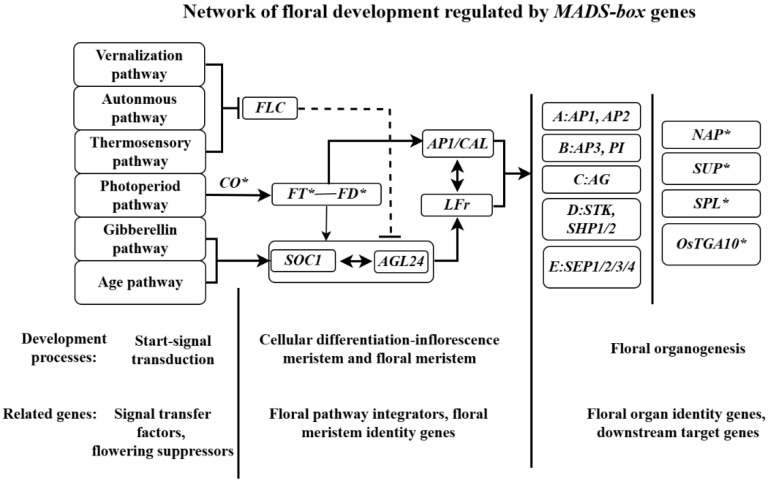
Relative MADS-box genes regulate floral development network, floral development processes, and related genes. → indicated acceleration; ─┤ inhibition; ─ represent this regulatory path has been studied; …… represent an unspecified regulatory path; * meant the gene was not a *MADS-box* gene. Genes unmarked were all *MADS-box* genes. *AP1*: *APETALA1*, *AP2*: *APETALA2*, *AP3*: *APETALA3*, *PI*: *PISTILLATA*; *AG*: *AGAMOUS*, *STK*: *SEEDSTICK*, *SHP1*: *SHATTERPROOF1*, *SHP2*: *SHATTERPROOF2*, *SEP*: *SEPALLATA*.

In recent years, research on the regulation of downstream target genes by *MADS-box* transcription factors has advanced rapidly, revealing their role in inducing floral organ formation through the activation of these targets. The first identified target gene of MADS-box proteins was *NAC-LIKE ACTIVATED BY AP3/PI* (*NAP*). *NAP* encodes a plant-specific NAC family protein and is induced by *AP3-PI* during petal and stamen development, controlling the transition from cell division to elongation [74]. Additionally, the expression of *NAP*-like genes increases during floral organ formation, fruit maturation, and senescence, suggesting their interaction with *MADS-box* genes in regulating the transition from flower to fruit development [75]. The restriction of boundaries between stamens and carpels requires the *SUPERMAN* (*SUP)* gene, which is regulated by *AP3*, *PI*, and *AG* genes at the boundaries of these floral whorls. Although the regulatory mechanism remains unclear, studies propose that *SUP* also inhibits the expression of B-class genes *AP3* and *PI* in the fourth whorl (carpels) and balances cell proliferation between the third and fourth whorls [76]. The AG protein can bind to a CArG-box-like sequence in the 3′UTR of the *SPOROCYTELESS* (*SPL*) gene, activating *SPL* transcription. Thus, *SPL*, as a direct target of *AG*, encodes a transcription factor that regulates ovule patterning and early microsporogenesis [77]. In rice, the E-class protein *OsMADS8* targets *OsTGA10*, a gene encoding a *bZIP* transcription factor that is preferentially expressed during stamen development. Mutations in *OsTGA10* result in male sterility, highlighting its critical role in tapetum development through interactions with known tapetum-related genes [78] (Figure 4). The VvMADS39 protein interacts with VvAGAMOUS in table grape, and their dimer is essential for integument development by activating and sustaining VvINO expression. Furthermore, the synergistic cooperation between VvMADS39 and associated proteins plays a crucial role in maintaining floral meristem identity, as well as supporting ovule and fruit development [79]. Investigating downstream target genes of *MADS*-*box* transcription factors provides a more comprehensive understanding of the floral development regulatory network. For example, microarray technology has identified 47 target genes of *AP3* and *PI*, with 11 being primarily or exclusively expressed in flowers. In *Aquilegia*, 7049 direct target genes of *AP3-3* have been identified [80]. The types and functions of downstream target genes of *MADS*-*box* transcription factors are crucial for comprehensively understanding the mechanisms of floral organ development and diversification. With advancements in biotechnology, from yeast two-hybrid systems to chromatin immunoprecipitation, the discovery of more target genes of *MADS-box* transcription factors will further refine the regulatory network of floral development.

## 4. Conclusions and Prospectives

In 1991, Coen and Meyerowitz conducted a landmark study on floral organ mutants in *Arabidopsis thaliana* and *Antirrhinum majus*, proposing the ABC model of floral organ development—a major milestone in plant developmental biology [81]. Most genes in this model (e.g., *AP1*, *AP2*, *AP3*, *PI*, and *AG*) belong to the *MADS*-*box* family of transcription factors. Their work revealed that behind the complex morphology of flowers lies a highly conserved “genetic toolkit” encoded by *MADS*-*box* genes. According to the ABC model, sepals are specified by class A genes alone; petals by the combined activity of class A and B genes; stamens by class B and C genes together; and carpels by class C genes alone. This framework provided a critical foundation for subsequent molecular studies on floral organ identity [82]. Further research has demonstrated that MADS-box proteins form specific homo- or heterodimers, which then assemble into higher-order quartet complexes. These complexes bind directly to CArG box motifs in the regulatory sequences of target genes [83]. This mechanism functions as a “molecular switch”, precisely activating or repressing downstream gene expression and thus elucidating the biochemical basis of the ABC model.

Over the decades of research on floral organ development, scientists have explored the intrinsic mechanisms underlying floral structure formation at the cellular, molecular, and genetic levels. While gene expression studies do not supersede morphological or anatomical approaches, the vast amount of genetic data has helped address questions hidden beneath phenotypic observations. Research at the genetic level has established a foundational framework for understanding the regulatory network of floral development (Figure 4), which continues to be refined with the discovery of additional regulatory nodes. Among these nodes, the *MADS-box* gene family has garnered significant attention as a major regulatory factor. With advancements in sequencing technologies and bioinformatics, an increasing number of plant species have been studied in depth through genomic or transcriptomic sequencing, revealing the roles of numerous members of the *MADS-box* gene family in floral organ development across different species. For example, overexpression of the *BpAP1* gene in *European white birch* leads to early flowering [84]. In the *Phalaenopsis orchid*, the B-class gene *PhalPI* plays a crucial role in regulating the development of lateral petals and the labellum [85]. Studies on the dichogamy of *Cyclocarya paliurus* flowers show that *CpAG* interacts with gibberellins (GA) to break bud dormancy and regulate floral initiation through the GA pathway [86]. In chrysanthemum, ectopic expression of the sunflower C-class gene *HAM59* results in male sterility and floral structure transformation, with stamens in disk florets converting into petal-like structures, creating a double-flower phenotype [87]. These studies represent only the tip of the iceberg regarding the *MADS-box* gene family, with many members yet to be explored. Future research will undoubtedly deepen our understanding of the complex regulation of floral organ development and enhance our comprehension of the higher-order functions of the *MADS-box* gene family. A key focus will be elucidating how *MADS-box* transcription factors form protein complexes and regulate target gene activity through epigenetic modifications, which will be a critical area of investigation in the study of the *MADS-box* gene family moving forward.

Through comparative genomic analysis of *Arabidopsis thaliana*, the phylogenetic relationships among the major evolutionary branches of the *MADS-box* gene family have been confirmed [7]. Determining these genes in different plant species will further aid in evaluating plant evolutionary relationships. *MADS-box* B- and C-class genes have been cloned from *Conifers* [88,89] and *Gnetales* [90], with studies indicating that these genes were present in the last common ancestor of angiosperms and gymnosperms. Their homologous genes were likely expressed in reproductive structures before the divergence of these two groups [38]. Although the homology of reproductive organs between gymnosperms and angiosperms remains unclear, *MADS-box* genes in gymnosperms will serve as powerful tools for further research into evolutionary systematics. Additionally, *MADS-box* genes have been identified in more distantly related plants such as mosses and ferns [54]. While these genes are not orthologous to those involved in floral structures, analyzing the functions of *MADS-box* genes across diverse plant groups is essential for constructing a comprehensive plant evolutionary tree. Although many *MADS-box* genes primarily function in reproductive development, some members of this family also play significant roles in vegetative growth and fruit development. For example, the *JOINTLESS* gene in tomatoes is associated with the abscission of leaves, flowers, and fruits [91]. In *Arabidopsis*, the *Arabidopsis nitrate regulated 1* (*ANR1*) gene controls nitrate regulation to modulate root growth [92]. *MADS-box* genes are widely expressed in the roots, stems, leaves, and embryos of angiosperms. By integrating these expression patterns and gene functions, we can elucidate the comprehensive role of *MADS-box* genes in plant morphological evolution and development.

In-depth research on *MADS*-*box* genes extends far beyond theoretical exploration, offering significant practical implications and widespread application value. In molecular breeding for crop improvement, many *MADS*-*box* genes (e.g., *SOC1*, *FLC*, *SVP*) function as key integrators within the floral transition pathway [93]. Modulating these genes through gene editing or conventional breeding allows the development of early- or late-flowering varieties adapted to specific environmental conditions, thereby helping address challenges posed by climate change or specific market requirements [94]. In the improvement of floral organ and fruit traits, direct regulation of class B or class C genes can modify floral architecture. For example, manipulating class B genes may lead to double-flowered phenotypes (with increased petal number) or facilitate the production of male sterile lines [95], while tuning class C gene activity can influence fruit development and seed formation [96,97]. Such approaches represent core technologies in the ornamental horticulture industry for creating novel floral morphologies and extending blooming periods. In studies of seed and fruit development, genes within the AG subfamily play direct roles in regulating carpel and fruit formation. Elucidating their mechanisms offers pathways to improve traits such as fruit size, fruit set rate, ripening timing, and seed characteristics, including the development of seedless varieties [98]. Moreover, *MADS*-*box* genes provide an excellent model system for investigating major evolutionary events, such as the origin of flowers and the radiation of angiosperms [99]. Comparative analyses of *MADS*-*box* gene function and expression across species can reveal fundamental mechanisms of plant evolution [100]. Furthermore, deciphering how *MADS*-*box* genes integrate environmental signals, such as vernalization and photoperiod, to control flowering time is essential for predicting plant phenological responses to global warming and for designing resilient crop varieties capable of thriving under future climate conditions [101].

Despite the significant progress achieved to date, research on the *MADS*-*box* gene family continues to hold promising and expansive prospects. Future studies will increasingly focus on elucidating the complex interaction networks between *MADS*-*box* genes and other transcription factors, epigenetic regulators (such as DNA methylation and histone modification), hormone signaling pathways (e.g., gibberellins and auxins), and non-coding RNAs [102,103,104]. Such integrated approaches will provide a more systematic understanding of the regulatory mechanisms controlling floral development. The application of single-cell sequencing and spatial transcriptomics will enable the mapping of *MADS-box* gene expression profiles and their dynamic changes at cellular resolution [105]. These technologies are expected to precisely uncover the roles of *MADS*-*box* genes in floral meristem initiation and the specification of floral organ primordia, potentially revealing novel cell types and more detailed regulatory mechanisms. While current knowledge is largely derived from a limited set of model plants, future research should expand functional investigations to economically important or evolutionarily significant species, such as orchids, cereal crops, and forest trees, to identify novel, species-specific genes and functions [106,107,108]. This expansion will open new avenues for precision breeding and trait engineering. Furthermore, synthetic biology approaches may allow the reprogramming of *MADS-box* gene expression combinations and regulatory circuits, potentially enabling the design of novel floral structures in heterologous systems. Such achievements would not only demonstrate considerable commercial value but also represent the ultimate test of our foundational knowledge of floral development.

## Figures and Tables

**Table 1:** MADS-box genes and their functions.

Gene	Function	Expression Pattern	Homologous Gene
*FLC*	Control of flowering time, flowering suppressor	Except shoot apex, widely expressed before flowering, download expression caused flowering	Cereal plants have no FLC homologous genes
*SVP*	Control of flowering time, flowering suppressor	Apical meristem of inflorescence, buds, leaves before flowering	Barley: BM1, BM9, HvVRT2; Wheat: TaVRT2
*SOC1*	Control of flowering time	Shoot apical meristem, leaves, flower buds	Brassica: LF, MF1, MF2
*CAL*	Floral meristem identity	Floral meristem	Paralogs of AP1
*FUL*	Control of flowering time, floral meri stemidentity, fruit development	Inflorescence meristem, ovules, cauline leaves	Paralogs of AP1
*AGL24*	Control of flowering time, flowering activator	Floral meristem	Paralogs of SVP
*AP1*	Floral meristem identity, A-class homeotic gene, regulated sepals and petals	Throughout floral meristem, whorls 1 and whorls 2 of floral organs	Snapdragon: SQUA, DEFH28; Rice: OsMADS14, OsMADS15, OsMADS18
*AP3*	B-class homeotic gene, regulated petals and stamens	Whorls 2 and whorls 3 of floral organs	Snapdragon: DEF, Petunia: PhGLO1/2, Rice: OsMADS16, Maize: Si1
*PI*	B-class homeotic gene, regulated petals and stamens	Whorls 2 and whorls 3 of floral organs	Snapdragon: GLO; Petunia: pMADS1,GP; Rice: OsMADS2, OsMADS4
*AG*	C-class homeotic gene, regulated stamens and carpels	Whorls 3 and whorls 4 of floral organs	Snapdragon: FAR, Petunia: pMADS3, Rice: OsMADS3, Maize: ZAG1
*SHP1/2*	D-class homeotic gene, fruit development and dehiscence	Ovules, valve margin, fruit dehiscence zone	Snapdragon: PLE; Petunia: FBP6
*STK*	D-class homeotic gene, regulated ovules development	Ovules	Petunia: FBP7, FBP11, Rice: OsMADS13, OsMADS21
*SEP1/2/3/4*	E-class homeotic gene, co-regulated floral development, activated B-and C class genes	SEP1/2: all whorls of floral organs; SEP3: whorls 2 and whorls 3; SEP4: whorls 1	Petunia: FBP2, Tomato: TM5, Rice: OsMADS1

Only those MADS-box genes related to floral development were mainly listed. Unless otherwise stated, the genes mentioned were from *Arabidopsis.*

## References

[1] Xu H.X., Meng D., Yang Q., Chen T., Qi M., Li X.Y., Ge H., Chen J.W. (2023). Sorbitol induces flower bud formation via the *MADS*-*box* transcription factor *EjCAL* in *loquat*. J. Integr. Plant Biol..

[2] Adhikari P.B., Kasahara R.D. (2024). An overview on *MADS-box* members in plants: A meta-review. Int. J. Mol. Sci..

[3] Chopy M., Cavallini-Speisser Q., Chambrier P., Morel P., Just J., Hugouvieux V., Rodrigues Bento S., Zubieta C., Vandenbussche M., Monniaux M. (2024). Cell layer–specific expression of the homeotic *MADS-box* transcription factor *PhDEF* contributes to modular petal morphogenesis in petunia. Plant Cell.

[4] Zhao H.B., Jian H.M., Wang Y., Wang G.Y., Zhou C.C., Jia H.J., Gao Z.S. (2019). Genome-wide identification and analysis of the *MADS-box* gene family and its potential role in fruit development and ripening in red bayberry (*Morella rubra*). Gene.

[5] Schwarz-Sommer Z., Huijser P., Nacken W., Saedler H., Sommer H. (1990). Genetic control of flower development by homeotic genes in *Antirrhinum majus*. Science.

[6] Heijmans K., Morel P., Vandenbussche M. (2012). *MADS-box* genes and floral development: The dark side. J. Exp. Bot..

[7] Alvarez-Buylla E.R., Pelaz S., Liljegren S.J., Gold S.E., Burgeff C., Ditta G.S., Ribas de Pouplana L., Martínez-Castilla L., Yanofsky M.F. (2000). An ancestral *MADS-box* gene duplication occurred before the divergence of plants and animals. Proc. Natl. Acad. Sci. USA.

[8] Lai X., Daher H., Galien A., Hugouvieux V., Zubieta C. (2019). Structural basis for plant *MADS* transcription factor oligomerization. Comput. Struct. Biotechnol. J..

[9] Bemer M., Heijmans K., Airoldi C., Davies B., Angenent G.C. (2010). An atlas of type I *MADS-box* gene expression during female gametophyte and seed development in *Arabidopsis*. Plant Physiol..

[10] Masiero S., Colombo L., Grini P.E., Schnittger A., Kater M.M. (2011). The emerging importance of type I *MADS-box* transcription factors for plant reproduction. Plant Cell.

[11] Kang I.H., Steffen J.G., Portereiko M.F., Lloyd A., Drews G.N. (2008). The AGL62 MADS domain protein regulates cellularization during endosperm development in *Arabidopsis*. Plant Cell.

[12] Zhang Y., Tang D., Lin X., Ding M., Tong Z. (2018). Genome-wide identification of *MADS-box* family genes in moso bamboo (*Phyllostachys edulis*) and a functional analysis of *PeMADS5* in flowering. BMC Plant Biol..

[13] Riechmann J.L., Meyerowitz E.M. (1997). *MADS* domain proteins in plant development. Biol. Chem..

[14] Lai X., Vega-Léon R., Hugouvieux V., Blanc-Mathieu R., van der Wal F., Lucas J., Silva C.S., Jourdain A., Muino J.M., Nanao M.H. (2021). The intervening domain is required for DNA-binding and functional identity of plant *MADS* transcription factors. Nat. Commun..

[15] Käppel S., Rümpler F., Theißen G. (2023). Cracking the Floral Quartet Code: How do multimers of MIKC^C^-type MADS-domain transcription factors recognize their target genes?. Int. J. Mol. Sci..

[16] Yoon J., Baek G., Pasriga R., Tun W., Min C.W., Kim S.T., Cho L.H., An G. (2023). Homeobox transcription factors OsZHD1 and OsZHD2 induce inflorescence meristem activity at floral transition in rice. Plant Cell Environ..

[17] Wang C., Yang X., Li G. (2021). Molecular insights into inflorescence meristem specification for yield potential in cereal crops. Int. J. Mol. Sci..

[18] Sharma N., Ruelens P., D’hauw M., Maggen T., Dochy N., Torfs S., Kaufmann K., Rohde A., Geuten K. (2017). A flowering locus C homolog is a vernalization-regulated repressor in *Brachypodium* and is cold regulated in wheat. Plant Physiol..

[19] Sharma N., Geuten K., Giri B.S., Varma A. (2020). The molecular mechanism of vernalization in *Arabidopsis* and cereals: Role of Flowering Locus C and its homologs. Physiol. Plant..

[20] Li Z., Jiang D., He Y. (2018). FRIGIDA establishes a local chromosomal environment for *FLOWERING LOCUS C* mRNA production. Nat. Plants.

[21] Li Z., Ou Y., Zhang Z., Li J., He Y. (2018). Brassinosteroid signaling recruits histone 3 lysine-27 demethylation activity to *FLOWERING LOCUS C* chromatin to inhibit the floral transition in *Arabidopsis*. Mol. Plant.

[22] Hong J.K., Park S.R., Suh E.J., Park J. (2020). Effects of overexpression of *Brassica rapa SHORT VEGETATIVE PHASE* gene on flowering time. Korean J. Breed. Sci..

[23] Liu X., Sun Z., Dong W., Wang Z., Zhang L. (2018). Expansion and functional divergence of the *SHORT VEGETATIVE PHASE* (*SVP*) genes in eudicots. Genome Biol. Evol..

[24] Xie L., Zhang Y., Wang K., Luo X., Xu D., Tian X., Li L., Ye X., Xia X., Li W. (2021). *TaVrt2*, an *SVP*-like gene, cooperates with *TaVrn1* to regulate vernalization-induced flowering in wheat. New Phytol..

[25] Liu X., Sun Z., Dong W., Wang Z., Zhang L. (2020). Transcriptome profiling reveals phase-specific gene expression in the developing barley inflorescence. Crop J..

[26] Wang Q., Liu W., Leung C.C., Tarté D.A., Gendron J.M. (2024). Plants distinguish different photoperiods to independently control seasonal flowering and growth. Science.

[27] Rehman S., Bahadur S., Xia W. (2023). An overview of floral regulatory genes in annual and perennial plants. Gene.

[28] Wang P., Su L., Cao L., Hu H., Wan H., Wu C., Zheng Y., Bao C., Liu X. (2024). AtSRT1 regulates flowering by regulating flowering integrators and energy signals in *Arabidopsis*. Plant Physiol. Biochem..

[29] Cai F., Jin X., Han L., Chen H., Shao C., Shi G., Bao M., Sun Y., Zhang J. (2024). AINTEGUMENTA-LIKE genes regulate reproductive growth and bud dormancy in *Platanus acerifolia*. Plant Cell Rep..

[30] Lee J., Oh M., Park H., Lee I. (2008). *SOC1* translocated to the nucleus by interaction with *AGL24* directly regulates *LEAFY*. Plant J..

[31] Torti S., Fornara F. (2012). *AGL24* acts in concert with *SOC1* and *FUL* during *Arabidopsis* floral transition. Plant Signal. Behav..

[32] Goslin K., Zheng B., Serrano-Mislata A., Rae L., Ryan P.T., Kwaśniewska K., Thomson B., Ó’Maoiléidigh D.S., Madueño F., Wellmer F. (2017). Transcription factor interplay between *LEAFY* and *APETALA1*/*CAULIFLOWER* during floral initiation. Plant Physiol..

[33] Pabón-Mora N., Ambrose B.A., Litt A. (2012). Poppy *APETALA1*/*FRUITFULL* orthologs control flowering time, branching, perianth identity, and fruit development. Plant Physiol..

[34] Sri T., Gupta B., Tyagi S., Singh A. (2020). Homeologs of *Brassica SOC1*, a central regulator of flowering time, are differentially regulated due to partitioning of evolutionarily conserved transcription factor binding sites in promoters. Mol. Phylogenetics Evol..

[35] Hill K., Wang H., Perry S.E. (2008). A transcriptional repression motif in the *MADS* factor *AGL15* is involved in recruitment of histone deacetylase complex components. Plant J..

[36] Agliassa C., Narayana R., Bertea C.M., Rodgers C.T., Maffei M.E. (2018). Reduction of the geomagnetic field delays *Arabidopsis thaliana* flowering time through downregulation of flowering-related genes. Bioelectromagnetics.

[37] Tan X.M., Li Y.R., Song M.R., Yuan L.N., Zhao Z.X., Liu Y., Meng Q., Huang X., Ma Y.Y., Xu Z.Q. (2025). The Molecular mechanism of interaction between *SEPALLATA3* and *APETALA1* in *Arabidopsis thaliana*. Plant Direct.

[38] Xu Y., Prunet N., Gan E.S., Wang Y., Stewart D., Wellmer F., Huang J., Yamaguchi N., Tatsumi Y., Kojima M. (2018). *SUPERMAN* regulates floral whorl boundaries through control of auxin biosynthesis. EMBO J..

[39] Kong L., Duan Y., Ye Y., Cai Z., Wang F., Qu X., Qiu R., Wu C., Wu W. (2019). Screening and analysis of proteins interacting with *OsMADS16* in rice (*Oryza sativa* L.). PLoS ONE.

[40] Otani M., Aoyagi K., Nakano M. (2020). Suppression of B function by chimeric repressor gene-silencing technology (CRES-T) reduces the petaloid tepal identity in transgenic *Lilium* sp.. PLoS ONE.

[41] Angent G.C., Colombo L. (1996). Molecular control of ovule development. Am. Ind. Hyg. Assoc. Q..

[42] Colombo L., Franken J., Koetje E., van Went J., Dons H.J.M., Angenent G.C., van Tunen A.J. (1995). The petunia MADS box gene *FBP11* determines ovule identity. Plant Cell.

[43] Immink R.G., Tonaco I.A., de Folter S., Shchennikova A., van Dijk A.D., Busscher-Lange J., Borst J.W., Angenent G.C. (2009). *SEPALLATA3*: The ‘glue’ for *MADS-box* transcription factor complex formation. Genome Biol..

[44] Pelaz S., Ditta G.S., Baumann E., Wisman E., Yanofsky M.F. (2000). B and C floral organ identity functions require *SEPALLATA MADS*-*box* genes. Nature.

[45] Gioppato H.A., Dornelas M.C. (2019). When Bs are better than As: The relationship between B-class *MADS-box* gene duplications and the diversification of perianth morphology. Trop. Plant Biol..

[46] Xiang L., Chen Y., Chen L., Fu X., Zhao K., Zhang J., Sun C. (2018). B and E *MADS-box* genes determine the perianth formation in *Cymbidium goeringii* Rchb.f. Physiol. Plant..

[47] Mandel M.A., Gustafson-Brown C., Savidge B., Yanofsky M.F. (1992). Molecular characterization of the *Arabidopsis* floral homeotic gene *APETALA1*. Nature.

[48] Gustafson-Brown C., Savidge B., Yanofsky M.F. (1994). Regulation of the *arabidopsis* floral homeotic gene *APETALA1*. Cell.

[49] Liu Z., Fei Y., Zhang K., Fang Z. (2019). Ectopic Expression of a Fagopyrum esculentum APETALA1 Ortholog only Rescues Sepal Development in *Arabidopsis ap1* Mutant. Int. J. Mol. Sci..

[50] Yang Q., Luo L., Jiao X., Chen X., Liu Y., Liu Z. (2024). APETALA2-like Floral Homeotic Protein Up-Regulating *FaesAP1_2* Gene Involved in Floral Development in Long-Homostyle Common Buckwheat. Int. J. Mol. Sci..

[51] Bowman J.L., Alvarez J., Weigel D., Meyerowitz E.M., Smyth D.R. (1993). Control of flower development in *Arabidopsis thaliana* by *APETALA1* and interacting genes. Development.

[52] Kater M.M., Dreni L., Colombo A.L. (2006). Functional conservation of *MADS*-*box* factors controlling floral organ identity in rice and *Arabidopsis*. J. Exp. Bot..

[53] Ma Y.Q., Pu Z.Q., Tan X.M., Meng Q., Zhang K.L., Yang L., Ma Y.Y., Huang X., Xu Z.Q. (2022). *SEPALLATA*-like genes of *Isatis indigotica* can affect the architecture of the inflorescences and the development of the floral organs. PeerJ.

[54] Ferrándiz C., Gu Q., Martienssen R., Yanofsky M.F. (2000). Redundant regulation of meristem identity and plant architecture by *FRUITFULL*, *APETALA1* and *CAULIFLOWER*. Development.

[55] Wagner D., Sablowski R.W., Meyerowitz E.M. (1999). Transcriptional activation of *APETALA1* by *LEAFY*. Science.

[56] Zhang S., Lu S., Yi S., Han H., Liu L., Zhang J., Bao M., Liu G. (2017). Functional conservation and divergence of five SEPALLATA-like genes from a basal eudicot tree, *Platanus acerifolia*. Planta.

[57] Song Q., Bari A., Li H., Chen L.L. (2020). Identification and analysis of micro-exons in *AP2/ERF* and *MADS* gene families. FEBS Open Bio.

[58] Dipp-Álvarez M., Cruz-Ramírez A. (2019). A Phylogenetic Study of the *ANT* Family Points to a *preANT* Gene as the ancestor of basal and *euANT* transcription factors in land plants. Front. Plant Sci..

[59] Xie W., Ding C., Hu H., Dong G., Zhang G., Qian Q., Ren D. (2022). Molecular events of rice *AP2*/*ERF* transcription factors. Int. J. Mol. Sci..

[60] Shannon S., Meeks-Wagner D.R. (1993). Genetic interactions that regulate inflorescence development in *Arabidopsis*. Plant Cell.

[61] Jofuku K.D., den Boer B.G., Van Montagu M., Okamuro J.K. (1994). Control of *Arabidopsis* flower and seed development by the homeotic gene *APETALA2*. Plant Cell.

[62] Schmid M., Uhlenhaut N.H., Godard F., Demar M., Bressan R., Weigel D., Lohmann J.U. (2003). Dissection of floral induction pathways using global expression analysis. Development.

[63] Aida M., Beis D., Heidstra R., Willemsen V., Blilou I., Galinha C., Nussaume L., Noh Y.S., Amasino R., Scheres B. (2004). The PLETHORA genes mediate patterning of the *Arabidopsis* root stem cell niche. Cell.

[64] Klucher K.M., Chow H., Reiser L., Fischer R.L. (1996). The *AINTEGUMENTA* gene of *Arabidopsis* required for ovule and female gametophyte development is related to the floral homeotic gene *APETALA2*. Plant Cell.

[65] Elliott R.C., Betzner A.S., Huttner E., Oakes M.P., Tucker W.Q., Gerentes D., Perez P., Smyth D.R. (1996). *AINTEGUMENTA*, an *APETALA2*-like gene of *Arabidopsis* with pleiotropic roles in ovule development and floral organ growth. Plant Cell.

[66] Zhao L.M., Kong H.Z., Leebens-Mack J.H., Kim S., Soltis P.S., Landherr L.L., Soltis D.E., de Pamphilis C.W., Ma H. (2005). The evolution of the *SEPALLATA* subfamily of *MADS*-*box* genes: A preangiosperm origin with multiple duplications throughout angiosperm history. Genetics.

[67] Goto K., Meyerowitz E.M. (1994). Function and regulation of the *Arabidopsis* floral homeotic gene *PISTILLATA*. Genes Dev..

[68] Fukui M., Futamura N., Mukai Y., Wang Y.Q., Nagao A., Shinohara K. (2001). Ancestral *MADS-box* genes in Sugi, *Cryptomeria japonica* D. Don (*Taxodiaceae*), homologous to the B function genes in *Angiosperms*. Plant Cell Physiol..

[69] Zhou L., Iqbal A., Yang M., Yang Y. (2025). Research progress on gene regulation of plant floral organogenesis. Genes.

[70] Takeda S., Hamamura Y., Sakamoto T., Kimura S., Aida M., Higashiyama T. (2022). Non-cell-autonomous regulation of petal initiation in *Arabidopsis thaliana*. Development.

[71] Pinyopich A., Ditta G.S., Savidge B., Liljegren S.J., Baumann E., Wisman E., Yanofsky M.F. (2003). Assessing the redundancy of *MADS*-*box* genes during carpel and ovule development. Nature.

[72] Rodríguez-Cazorla E., Ripoll J.J., Ortuño-Miquel S., Martínez-Laborda A., Vera A. (2020). Dissection of the *Arabidopsis HUA*-*PEP* gene activity reveals that ovule fate specification requires restriction of the floral A-function. New Phytol..

[73] Hussin S.H., Wang H., Tang S., Zhi H., Tang C., Zhang W., Jia G., Diao X. (2021). *SiMADS34*, an E-class *MADS*-*box* transcription factor, regulates inflorescence architecture and grain yield in *Setaria italica*. Plant Mol. Biol..

[74] Jiang Y., Wang M., Zhang R., Xie J., Duan X., Shan H., Xu G., Kong H. (2020). Identification of the target genes of *AqAPETALA3*-3(*AqAP3*-3) in *Aquilegia coerulea* (*Ranunculaceae*) helps understand the molecular bases of the conserved and nonconserved features of petals. New Phytol..

[75] Huang F., Zhang Y., Hou X. (2020). *BcAP3*, a *MADS-box* gene, controls stamen development and male sterility in Pak-choi (*Brassica rapa* ssp. *chinensis*). Gene.

[76] Wan S., Liang B., Yang L., Hu W., Kuang L., Song J., Xie J., Huang Y., Liu D., Liu Y. (2023). The *MADS-box* family gene *PtrANR1* encodes a transcription activator promoting root growth and enhancing plant tolerance to drought stress. Plant Cell Rep..

[77] Mao T., Wang X., Gao H., Gong Z., Liu R., Jiang N., Zhang Y., Zhang H., Guo X., Yu C. (2023). Ectopic Expression of *MADS-box* transcription factor *VvAGL12* from grape promotes early flowering, plant growth, and production by regulating cell-wall architecture in *Arabidopsis*. Genes.

[78] Prunet N. (2019). My favourite flowering image: An *Arabidopsis* inflorescence expressing fluorescent reporters for the *APETALA3* and *SUPERMAN* genes. J. Exp. Bot..

[79] Zhang S., Yao J., Wang L., Wu N., van Nocker S., Li Z., Gao M., Wang X. (2022). Role of grapevine *SEPALLATA*-related *MADS-box* gene *VvMADS39* in flower and ovule development. Plant J..

[80] Uemura A., Yamaguchi N., Xu Y., Wee W., Ichihashi Y., Suzuki T., Shibata A., Shirasu K., Ito T. (2018). Regulation of floral meri-stem activity through the interaction of *AGAMOUS*, *SUPERMAN*, and *CLAVATA3* in *Arabidopsis*. Plant Reprod..

[81] Coen E.S., Meyerowitz E.M. (1991). The war of the whorls: Genetic interactions controlling flower development. Nature.

[82] Cozzolino S., Widmer A. (2005). Orchid diversity: An evolutionary consequence of deception?. Trends Ecol. Evol..

[83] Sun J., Liu Y., Zheng Y., Xue Y., Fan Y., Ma X., Ji Y., Liu G., Zhang X., Li Y. (2024). The *MADS-box* transcription factor *GmFULc* promotes *GmZTL4* gene transcription to modulate maturity in soybean. J. Integr. Plant Biol..

[84] Chen Z.S., Liu X.F., Wang D.H., Chen R., Zhang X.L., Xu Z.H., Bai S.N. (2018). Transcription factor *OsTGA10* is a target of the *MADS* protein *OsMADS8* and is required for tapetum development. Plant Physiol..

[85] Shulga O.A., Mitiouchkina T.Y., Shchennikova A.V., Skryabin K.G., Dolgov S.V. (2015). Chrysanthemum modification via ectopic expression of sunflower *MADS-box* gene *HAM59*. Acta Hortic..

[86] Mouradov A., Glassick T.V., Hamdorf B.A., Murphy L.C., Marla S.S., Yang Y., Teasdale R.D. (1998). Family of *MADS-box* genes expressed early in male and female reproductive structures of *Monterey pine*. Plant Physiol..

[87] Winter K.U., Becker A., Münster T., Kim J.T., Saedler H., Theissen G. (1999). *MADS-box* genes reveal that gnetophytes are more closely related to conifers than to flowering plants. Proc. Natl. Acad. Sci. USA.

[88] Thangavel G., Nayar S. (2018). A survey of *MIKC* type *MADS-box* genes in non-seed plants: Algae, bryophytes, lycophytes and ferns. Front. Plant Sci..

[89] Mao L., Begum D., Chuang H.W., Budiman M.A., Szymkowiak E.J., Irish E.E., Wing R.A. (2000). *JOINTLESS* is a *MADS-box* gene controlling tomato flower abscissionzone development. Nature.

[90] Gan Y., Bernreiter A., Filleur S., Abram B., Forde B.G. (2012). Overexpressing the *ANR1 MADS-box* gene in transgenic plants provides new insights into its role in the nitrate regulation of root development. Plant Cell Physiol..

[91] Xing M., Li H., Liu G., Zhu B., Zhu H., Grierson D., Luo Y., Fu D. (2022). A *MADS-box* transcription factor, *SlMADS1*, interacts with *SlMACROCALYX* to regulate tomato sepal growth. Plant Sci..

[92] Lin J.H., Yu L.H., Xiang C.B. (2020). *ARABIDOPSIS NITRATE REGULATED 1* acts as a negative modulator of seed germination by activating *ABI3* expression. New Phytol..

[93] Kumar K., Srivastava H., Das A., Tribhuvan K.U., Durgesh K., Joshi R., Sevanthi A.M., Jain P.K., Singh N.K., Gaikwad K. (2021). Identification and characterization of *MADS-box* gene family in pigeonpea for their role during floral transition. 3 Biotech.

[94] Zhang C., Shen J., Wang C., Wang Z., Guo L., Hou X. (2022). Characterization of *PsmiR319* during flower development in early- and late-flowering tree peonies cultivars. Plant Signal. Behav..

[95] Nakatsuka T., Saito M., Yamada E., Fujita K., Yamagishi N., Yoshikawa N., Nishihara M. (2015). Isolation and characterization of the C-class *MADS-box* gene involved in the formation of double flowers in Japanese gentian. BMC Plant Biol..

[96] Zhao J., Gong P., Liu H., Zhang M., He C. (2021). Multiple and integrated functions of floral C-class *MADS-box* genes in flower and fruit development of *Physalis floridana*. Plant Mol. Biol..

[97] Garceau D.C., Batson M.K., Pan I.L. (2017). Variations on a theme in fruit development: The PLE lineage of *MADS-box* genes in tomato (*TAGL1*) and other species. Planta.

[98] Zhong S., Yang H., Guan J., Shen J., Ren T., Li Z., Tan F., Li Q., Luo P. (2022). Characterization of the *MADS-box* gene family in *Akebia trifoliata* and their evolutionary events in *Angiosperms*. Genes.

[99] Zhao D., Chen Z., Xu L., Zhang L., Zou Q. (2021). Genome-wide analysis of the *MADS-box* gene family in Maize: Gene structure, evolution, and relationships. Genes.

[100] Preston J.C., Fjellheim S. (2022). Flowering time runs hot and cold. Plant Physiol..

[101] Feng Y.Y., Du H., Huang K.Y., Ran J.H., Wang X.Q. (2024). Reciprocal expression of *MADS-box* genes and DNA methylation reconfiguration initiate bisexual cones in spruce. Commun. Biol..

[102] Zhang S.L., Wu Y., Zhang X.H., Feng X., Wu H.L., Zhou B.J., Zhang Y.Q., Cao M., Hou Z.X. (2024). Characterization of the MIKC^C^-type *MADS-box* gene family in blueberry and its possible mechanism for regulating flowering in response to the chilling requirement. Planta.

[103] Li N., Huang B., Tang N., Jian W., Zou J., Chen J., Cao H., Habib S., Dong X., Wei W. (2017). The *MADS-box* gene *SlMBP21* regulates sepal size mediated by ethylene and auxin in tomato. Plant Cell Physiol..

[104] Dai Y., Zhang S., Guan J., Wang S., Zhang H., Li G., Sun R., Li F., Zhang S. (2024). Single-cell transcriptomic analysis of flowering regulation and vernalization in Chinese cabbage shoot apex. Hortic. Res..

[105] Wang Y., Li Y., Yan X., Ding L., Shen L., Yu H. (2020). Characterization of C- and D-class *MADS-box* genes in *Orchids*. Plant Physiol..

[106] Zuo Z.W., Zhang Z.H., Huang D.R., Fan Y.Y., Yu S.B., Zhuang J.Y., Zhu Y.J. (2021). Control of thousand-grain weight by *OsMADS56* in rice. Int. J. Mol. Sci..

[107] Zhou P., Qu Y., Wang Z., Huang B., Wen Q., Xin Y., Ni Z., Xu L. (2023). Gene structural specificity and expression of *MADS-box* gene family in *Camellia chekiangoleosa*. Int. J. Mol. Sci..

[108] Yu Y., Chu X., Ma X., Hu Z., Wang M., Li J., Yin H. (2024). Genome-wide analysis of *MADS-box* gene family reveals *CjSTK* as a key regulator of seed abortion in *Camellia japonica*. Int. J. Mol. Sci..

